# Sleep Deprivation Modifies Noise-Induced Cochlear Injury Related to the Stress Hormone and Autophagy in Female Mice

**DOI:** 10.3389/fnins.2019.01297

**Published:** 2019-11-29

**Authors:** Pengjun Li, Dan Bing, Sumei Wang, Jin Chen, Zhihui Du, Yanbo Sun, Fan Qi, Yingmiao Zhang, Hanqi Chu

**Affiliations:** ^1^Department of Otolaryngology-Head and Neck Surgery, Tongji Hospital, Tongji Medical College, Huazhong University of Science and Technology, Wuhan, China; ^2^Department of Occupational and Environmental Health, School of Public Health, Tongji Medical College, Huazhong University of Science and Technology, Wuhan, China; ^3^Key Laboratory of Environment and Health, Ministry of Education & Ministry of Environmental Protection, State Key Laboratory of Environmental Health (Incubating), School of Public Health, Tongji Medical College, Huazhong University of Science and Technology, Wuhan, China; ^4^Department of Clinical Immunology, Tongji Hospital, Tongji Medical College, Huazhong University of Sciences and Technology, Wuhan, China

**Keywords:** sleep deprivation, noise, hidden hearing loss, synaptic ribbon, hair cell protection, corticosterone, autophagy, apoptosis

## Abstract

A lack of sleep is linked with a range of inner ear diseases, including hearing loss and tinnitus. Here, we used a mouse model to investigate the effects of sleep deprivation (SD) on noise vulnerability, and explored the mechanisms that might be involved *in vitro*, focusing particularly corticosterone levels and autophagic flux in cells. Female BALB/c mice were divided into six groups [control, acoustic trauma (AT)-alone, 1 day (d) SD-alone, 1d SD pre-AT, 5d SD-alone, and 5d SD pre-AT]. Cochlear damage was then assessed by analyzing auditory brainstem response (ABR), and by counting outer hair cells (OHCs) and the synaptic ribbons of inner hair cells (IHCs). In addition, we measured levels of serum corticosterone and autophagy protein expression in the basilar membranes by ELISA kits, and western blotting, respectively. We found that SD-alone temporarily elevated ABR wave I amplitude, but had no permanent effect on hearing level or IHC ribbon numbers. Combined with AT, the number of synaptic ribbons in the 1d SD pre-AT group was significantly higher than that in the AT-alone group, whereas the 5d SD pre-AT group showed more severe synaptopathy, and a greater loss of OHCs after 2 weeks than the other experimental groups exposed to noise. Correspondingly, the levels of corticosterone in the AT-alone group were higher than those of the 1d SD pre-AT group, but lower than those of the 5d SD pre-AT group. The 1d SD pre-AT group showed a marked elevation in the expression of microtubule-associated protein 1 light chain 3B (LC3B), whereas the AT-alone group exhibited only a mild increase. In contrast, the levels of LC3B did not change in the 5d SD pre-AT group. Experiments with HEI-OC-1 cells and cochlear basilar membrane cultures showed that high-concentrations of dexamethasone, and the inhibition of autophagy, aggravated cellular apoptosis induced by oxidative stress. In conclusion, noise-induced synaptopathy and hair cell loss can be mitigated by preceding 1d SD, but will be aggravated by preceding 5d SD. These findings may be attributable to corticosterone levels and the extent of autophagy.

## Introduction

Sleep is indispensable for the homeostasis of organisms, and is closely associated with circadian rhythms (Goel et al., 2013) and hormone levels (Mônico-Neto et al., 2015). The number of people suffering from sleep disorders is increasing, particularly those who only sleep for a few hours each night (Foster and Kreitzman, 2014). This represents a major issue because sleep disorders are known to increase the risk of some diseases, including obesity (Van Cauter et al., 2008), diabetes (Chao et al., 2011), cancer (Haus and Smolensky, 2013), cardiovascular diseases (Chaput et al., 2013; McAlpine et al., 2019), Alzheimer Disease, and dementia (Pistollato et al., 2016). Sleep interventions can serve as a treatment for depression (Howland, 2011). However, sleep deprivation (SD) can also be detrimental to the auditory system. In clinical practice, it is common for SD to aggravate the perception of tinnitus (Pan et al., 2015; Bhatt et al., 2017). Furthermore, over 50% of sudden deafness patients had previously experienced SD (Yang et al., 2015). From another aspect, both animal and human studies have demonstrated that noise could alter the auditory brainstem response (ABR) and produce tinnitus without affecting the function of the outer hair cells (Bramhall et al., 2017; Liberman and Kujawa, 2017). Tinnitus may arise from excessive activity in the auditory center in response to reduced peripheral input (Auerbach et al., 2014). Studies have also reported an increased risk of tinnitus in humans when there was a reduction in the wave I amplitude of ABR (Bramhall et al., 2018). Animal studies have shown that reductions in wave I amplitude are associated with decreased number of ribbon synapses (Bing et al., 2015). However, whether SD alone causes cochlear injury, or whether SD modifies susceptibility to noise, has yet to be elucidated.

In humans, just 1 day (1d) of SD was enough to cause an increase in cortisol levels (Leproult et al., 1997). In mice, successive paradoxical SD was shown to activate the hypothalamic-pituitary-adrenal (HPA) axis, and regulate the plasma levels of corticosterone, in a manner that was dependent upon the length of SD (Gonzalez-Castañeda et al., 2016). Corticosterone is the major corticosteroid in rodents; the elevation of this hormone activates glucocorticoid receptors, and interferes with metabolism, including the metabolic activity of the autophagy-lysosomal system (Bonaldo and Sandri, 2012). It is important that a highly regulated system maintains synapse integrity and synaptic function for presynaptic boutons (Hoffmann et al., 2019). Previous studies have shown that corticosterone accelerates glutamatergic transmission between nerve cells, which quickly, and reversibly, increases the frequency of miniature excitatory post-synaptic currents (Karst et al., 2010). We therefore hypothesized that both corticosterone and autophagy play important roles in preserving synaptic function. Furthermore, corticosterone level and autophagy have been reported to be strongly associated with noise-induced cochlear injury. Previous research has demonstrated the existence of corticosterone receptors in the inner ear, and in particular, the organ of Corti, and the spiral ligament (Rarey et al., 1993; Tahera et al., 2006). High levels of corticosterone trigger a protective effect on cochlear outer hair cells (OHCs) (Wang and Liberman, 2002; Takemura et al., 2004; Meltser et al., 2009; Meltser and Canlon, 2011). Moreover, antagonizing corticosterone receptors was previously shown to attenuate sound-induced long-term deficits in the auditory nerve (Singer et al., 2018). On the other hand, the activation of autophagy in the cochlea protects hair cells from acoustic trauma by clearing damaged cellular constituents, including reactive oxygen species (Yuan et al., 2015; Ye et al., 2019).

In this study, we investigated whether SD modifies noise-susceptibility in the cochlea of female mice, and investigated the effects of SD on the regulation of circulating corticosterone levels and autophagy. We also carried out experiments with hair cells (HCs) and HEI-OC-1 cells in order to investigate the potential role of corticosterone levels and autophagy in cochlea damage.

## Materials and Methods

### Animals and Experimental Procedure

Female BALB/C mice (weighing 17–20 g and aged 8–9 weeks) were used for *in vivo* studies. The *in vitro* experiments involved post-natal day 3 rats. Animals were purchased from Beijing Vital River Laboratory Animal Technology Co., Ltd. Animal care and experimental protocols were approved by the Animal Research and Ethics Committee of Tongji Medical College, Huazhong University of Science and Technology, China.

**FIGURE 1:**
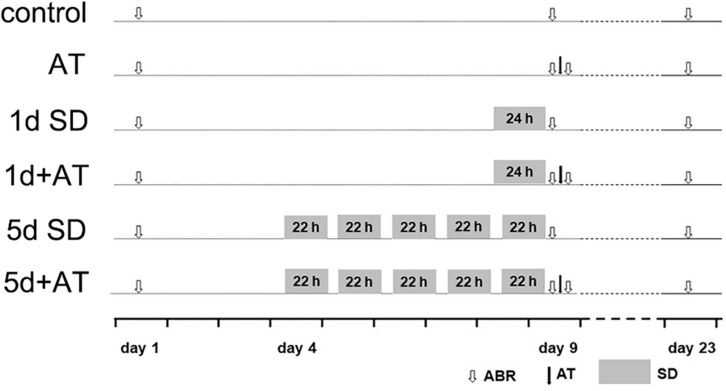
Experimental procedure for animals. Experimental timeline for six groups: control (sham SD followed by sham AT), AT (sham SD followed by AT), 1d SD (1d SD followed by sham AT), 1d + AT (1d SD followed by AT), 5d SD (5d SD followed by sham AT), 5d + AT (5d SD followed by AT). ABR, auditory brainstem response recording.

For the *in vivo* study, the animals were divided into six groups, including control (*n* = 36 mice), acoustic trauma-alone (AT, *n* = 36 mice), 1 day SD-alone (1d SD, *n* = 34 mice), 1d SD pre-AT (1d + AT, *n* = 38 mice), 5d SD-alone (5d SD, *n* = 35 mice), and 5d SD pre-AT (5d + AT, *n* = 37 mice). Animals were then subjected to a range of different procedures, as illustrated in Figure 1. Auditory brainstem response (ABR) thresholds were tested at baseline (pre-SD), immediately after SD (post-SD), immediately after AT (post-AT), and 2 weeks after AT (post-2w). For hearing measurements, and acoustic overexposures, animals were anesthetized with the mixture of chlorpromazine hydrochloride at a dose of 20 mg/kg body weight (Harvest Pharmaceutical Co., Ltd., Shanghai, China), and ketamine hydrochloride at a dose of 120 mg/kg body weight (Gutian Pharma Co., Ltd., Ningde, China). Two weeks after AT, or sham AT, the animals were sacrificed. Tissues were then collected and prepared for analysis.

### Sleep Deprivation

We adopted the multiple-platform water environmental method as a model for SD, as described previously (Hirotsu et al., 2012; Gonzalez-Castañeda et al., 2016). First, the mice were settled in a polypropylene container (40 cm × 30 cm × 15 cm) containing five circular platforms (3 cm in diameter). The water level in each container was 2 cm in depth from the bottom of the container, and 0.5 cm below the standing surface of the platforms. The mice had free access to food and water, and were able to leap between platforms. Once a paradoxical sleep episode began, the close contact with water forced the animals to wake up. We designed two patterns of SD: continuous 1 day and segmental 5 days. Across the 5 days, each day consisted of 22 h SD and 2 h sleep-free. Sham SD, and sleep-free animals, were maintained in the same room and the same container with wood shavings instead of water.

### Acoustic Over-Exposure

Animals were exposed to broad band noise (8–16 kHz) under anesthesia at 105 dB (SPL) for 2 h. During exposure, the mice were placed in small compartments within the large cage. The cage was then placed immediately below the horn. Noise levels were calibrated at the beginning of each exposure. The difference in noise level between each compartment was less than 1dB. The whole device was placed in a small, reverberant chamber. Sham AT animals were placed in the same cage below the horn but without sound.

### Assessment of Auditory Function

Hearing threshold was assessed by click- and tone burst-ABR recordings. Anesthetized mice were allowed to lie prone on the heating plate to maintain body temperature. A TDT system III (Tucker-Davis Technologies, Alachua, FL, United States) was used to generate stimuli and record trigger signals. Generated tone burst stimuli (at 8, 16, 24, and 32 kHz) were delivered into the external auditory canal of each mouse through an electrostatic speaker that was placed next to the head. The TDT system filtered the evoked potentials between 100 and 3000 Hz and averaged it for 512 times. The highest stimulus intensity was 90 dB; the threshold vale was determined by reducing the intensity by 10dB to identify the lowest sound level that just elicited a repeatable wave. The ABR test was carried out on 6 ears in the control group, 6 ears in the AT group, 4 ears in the 1d SD group, 8 ears in the 1d + AT group, 5 ears in the 5d SD group, and 7 ears in the 5d + AT group.

ABR wave I amplitude was analyzed as described previously (Bing et al., 2015); the amplitude was defined as the vertical distance from the starting negative (n) peak to the following positive (p) peak. One of researcher measured the ABR in mice. The interpretation and statistics relating to the threshold and the wave amplitude were performed by another researcher who was blinded to the groupings.

### Cell Culture, Tissue Culture, and Drug Administration

HEI-OC-1 cells were cultured at 33°C with 10% CO_2_ in DMEM containing 1.0 g/L of glucose, 10% FBS, and 1% N-2 (Gibco, New York, NY, United States). The middle cochlea basilar membranes were then dissected from P3 rats and laid onto a glass sheet (coated with ornithine and laminin) that had been placed in a dish. The HEI-OC-1 cells, and the HCs, were cultured with different concentrations of dexamethasone (0.5, 5 μM) for 24 h, or with 0.5 nM rapamycin, or 5 mM 3-Ma, for 6 h before exposure to H_2_O_2_. HEI-OC-1 cells were then treated with H_2_O_2_ (2, 5, and 10 mM) while the HCs were treated with H_2_O_2_ (0.5, 0.8, 1 mM) for 1 h. Both cell types were then allowed to recover in culture medium for another 24 h, together with corresponding concentrations of dexamethasone, rapamycin, or 3-MA. The dexamethasone, rapamycin, and 3-MA were all obtained from Med Chem Express (NJ, United States).

### Corticosterone Assay

To measure serum corticosterone levels, we anesthetized the animals and collected blood samples by removing an eyeball at two time points (post-AT and post-2W). Blood samples were always collected at a specific time (4–6 PM). Blood samples were allowed to stand for 60 min at room temperature, and then centrifuged at 5000 rpm for 20 min at 4°C. Serum was then collected and stored at −80°C to await corticosterone assay. The serum corticosterone level was determined by an AssayMax corticosterone ELISA kit (AssayPro, St. Charles, United States).

### Immunohistochemistry

Immunohistochemistry was used to determine OHC loss and the number of inner hair cell synaptic ribbons in animal tissues, and to demonstrate the localization of LC3B in HEI-OC-1 cells. Animals were decapitated after blood collection and the cochleae were isolated and fixed overnight by immersion in 4% paraformaldehyde. Samples were then decalcified for 2 days in 10% sodium EDTA. Basilar membranes were then carefully dissected, as described previously (Zhu et al., 2013). HEI-OC-1 cells were fixed in 100% cold methyl alcohol at −20°C for 20 min.

Basilar membranes, or HEI-OC-1 cells, were then permeabilized for 20 min with 0.3% Triton X-100/PBS, washed with PBS, blocked for 60 min with 10% goat serum/PBS, and incubated overnight with anti-CTBP-2 (1:150; Abcam, Cambridge, United Kingdom) or anti-LC3B (1:100; Abcam, Cambridge, United Kingdom) at 4°C. The next day, the membranes and the cells were washed with PBS and incubated for 1 h with a secondary antibody (1:400; Multi sciences, Hangzhou, China). Membranes the cells were then washed twice with PBS and nuclei stained with DAPI (Servicebio, Wuhan, China). Next, the basilar membrane and cells were re-washed with PBS, adhered to glass slides, and covered with coverslips. Samples were then observed under a fluorescence microscope (400×). Fluorescent images were acquired by first detecting specific fluorescence (green), and then by continuously scanning the membranes until the green fluorescence disappeared. In the middle of each region of the cochlea, we counted the ribbons and OHCs corresponding to 15 inner hair cells. Fluorescent images of the membranes were collected by one researcher alone, while ribbons and hair cells were counted by another researcher who was blinded to the grouping. OHCs and ribbons were counted for all cochlear turns from at least 4 cochleae from each group.

Cultured basilar membranes were fixed for 40 min in 4% paraformaldehyde, permeabilized for 20 min with 0.3% Triton X-100/PBS, and then stained with 5 μg/ml of phalloidin-FITC (Sigma-Aldrich, St. Louis, MO, United States).

### Immunoblotting

Immunoblotting was performed at 2 timepoints (post-AT and post-2W). At each timepoint, the cochleae were first dissected, immersed in cold PBS. Immediately after immersion, the basilar membranes were carefully dissected for immunoblotting.

We used immunoblotting to detect the expression levels of autophagy-related proteins (LC3B, p62) in the cochlear basilar membranes. For each experimental group, we analyzed 12 cochleae from 6 mice. The tissues were homogenized in RIPA lysis buffer (Servicebio, Wuhan, China) containing protease inhibitors phenylmethanesulfonyl fluoride (PMSF) cocktail. The homogenates were then lysed by ultrasound, and then left to stand for 30 min on ice. Total protein was then collected by centrifugation, and the concentration of protein extracts determined using a bicinchoninic acid kit (Servicebio, Wuhan, China). Protein extracts were then separated on an SDS-PAGE gel (p62: 10%; LC3B: 15%) and then transferred to PVDF membranes. Membranes were first blocked for 1 h with 3% BSA/TBST, and then incubated overnight with several primary antibodies: LC3B (1:1000; Abcam, Cambridge, United Kingdom), p62 (1:1000; Abcam, Cambridge, United Kingdom), and beta-actin (1:1000; Servicebio, Wuhan, China). The following day, membranes were washed with TBST and incubated for 1 h with HRP-labeled Goat Anti-Rabbit IgG (H + L) (Servicebio, Wuhan, China). Next, the membranes were re-washed and immersed in electro-chemi-luminescence medium (Servicebio, Wuhan, China), and finally visualized with an E-Gel Imager. HEI-OC-1 cells were collected from a culture plate and homogenized. The remaining steps were the same as those used for cochlear tissues.

### Electron Microscopy

HEI-OC-1 cells were collected and fixed in 2.5% glutaraldehyde (Servicebio, Wuhan, China) for 24 h, and then in 1% osmic acid for 2 h. Cells were then dehydrated by a descending series of acetone gradients, embedded in 812 araldite, torrefied at 37°C for 24 h and 60°C for 48 h, and finally sectioned at a thickness of 60–80 nm by an ultrathin slicer. The sections were then stained with alcoholic uranyl acetate and alkaline lead citrate, washed with distilled water, and finally observed with a transmission electron microscope.

### Flow Cytometry

Apoptosis was evaluated using an Annexin V-FITC/PI kit (KeyGen BioTECH, Nanjing, China). After treatment with a range of drugs, HEI-OC-1 cells were trypsinized, centrifuged at 2000 rpm for 5 min, and then washed twice with PBS. The cells obtained from one hole of a six-hole plate were then resuspended in 500 μl of binding buffer. Annexin V-FITC, and propidium iodide, were the added to the cell suspension and gently mixed. The cells were then incubated for 15 min at room temperature in the dark and finally analyzed by flow cytometry.

### Statistical Analysis

Data are presented as means ± standard error of the mean (S.E.M.) or means ± standard deviation (S.D.). N represents the number animals per experimental group. The statistical significance of differences between groups was assessed by the Student’s *t*-test, 1-way analysis of variance (ANOVA), or 2-way ANOVA. *P*-values are presented as ^∗^*P* < 0.05; ^∗∗^*P* < 0.01; ^∗∗∗^*P* < 0.001, or n.s. (not significant).

## Results

### SD Had No Effect on Hearing Threshold but Temporarily Elevated the ABR Wave I Amplitude

**FIGURE 2:**
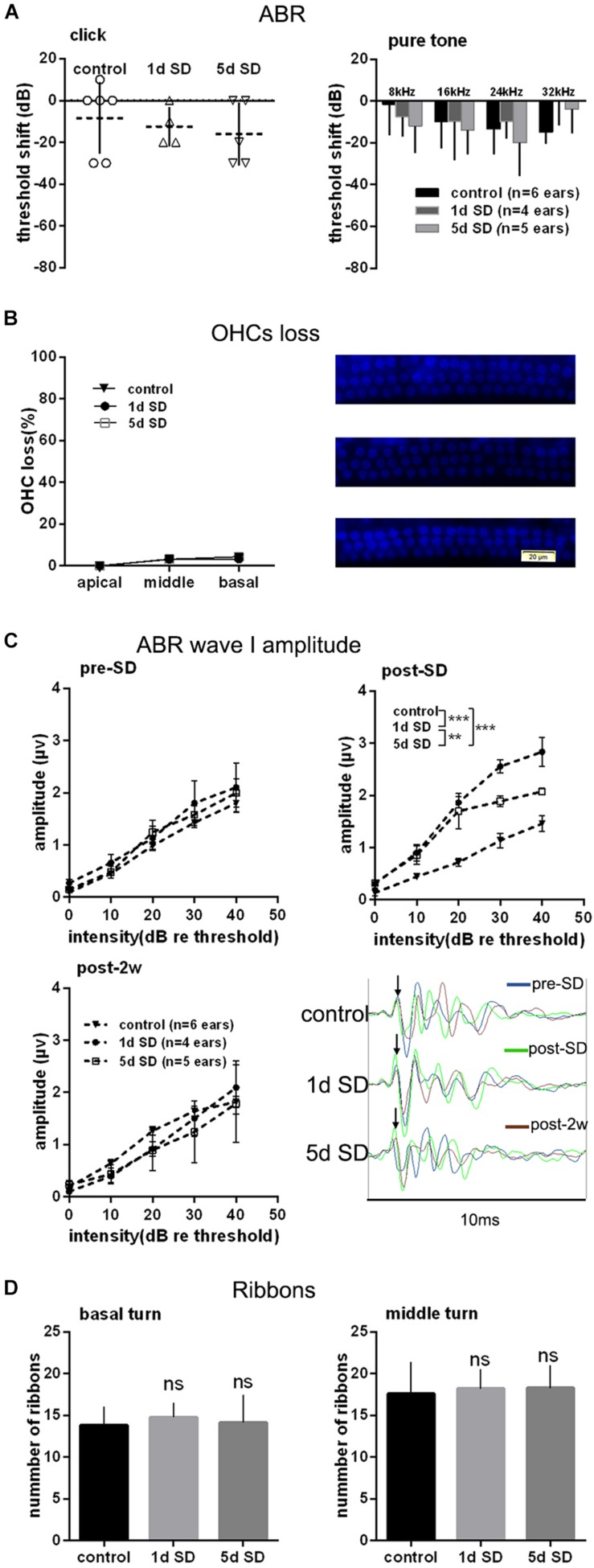
Audiometric threshold shifts, quantification of outer hair cells (OHCs) and inner hair cell (IHC) ribbons at post-2w, and ABR wave I amplitude for SD groups and control group. **(A)** Mean (transverse line) ± S.D. and individual ear audiometric threshold shift (dB) from baseline (pre-SD) determined from click and pure tone evoked ABRs for SD groups. The threshold shifts measured by click and pure tone stimulus were not statistically different between SD groups (click: one-way ANOVA, *F* = 362, *P* = 0.704; pure tone: two-way ANOVA, *F* = 0.917, *P* = 0.407 for effects of SD). **(B)** Mean ± S.D. OHCs loss percentage in the basal, middle and apical cochlear turns, and DAPI labeling of OHCs nucleus (blue) in the basal turn for both SD groups and control group. No significant difference was observed between SD groups and control group (two-way ANONA, *F* = 0.312, *P* = 0.735 for effects of SD). **(C)** Mean ± S.E.M. ABR wave I amplitude growth function for SD groups and control group. The baseline (pre-SD) wave I amplitudes were not significantly different among three groups (two-way ANOVA, *F* = 1.631, *P* = 0.205 for effects of SD), and the wave I amplitudes increased immediately after SD (post-SD) (two-way ANOVA, *F* = 37.853, *P* = 0.000; control vs. 1d SD group: two-way ANOVA, *P* = 0.000, control vs. 5d SD group: two-way ANOVA, *P* = 0.000, 1d SD vs. 5d SD group: two-way ANOVA, *P* = 0.001, see Table 1). After 2 weeks, the wave I amplitudes for both SD groups reduced to baseline (two-way ANOVA, *F* = 0.460, *P* = 0.634). **(D)** Mean ± S.D. IHC ribbons in the basal and middle cochlear turn for all SD groups at post-2w. No significant difference was detected between SD groups and control group for the basal turn (one-way ANONA, *F* = 1.663, *P* = 0.194) and middle turn (one-way ANONA, *F* = 0.558, *P* = 0.574).

To test the effect of SD on the auditory system, we examined the audiometric threshold shifts for different SD groups. As shown in Figure 2A, there were no significant differences in terms of hearing thresholds for click and pure tones stimuli, as measured by ABR between the SD groups and the control group. Similarly, there were no statistically significant differences in the loss of OHCs loss when compared across the control group, the 1d SD group, and the 5d SD group (Figure 2B).

**TABLE 1:** *Post hoc* comparisons (Student’s *t*-tests with Bonferroni adjustment) of ABR wave I amplitude evoked by noise burst stimuli at post-SD.

	**0**	**10**	**20**	**30**	**40**
**Intensity (dB re threshold)**					
Control vs. 1d SD	n.s.	n.s.	^∗∗^	^∗∗∗^	^∗∗^
Control vs. 5d SD	n.s.	n.s.	^∗^	^∗∗^	n.s.
1d SD vs. 5d SD	n.s.	n.s.	n.s.	^∗^	n.s.

*^∗^p < 0.05; ^∗∗^p < 0.01; ^∗∗∗^p < 0.001; n.s., not significant.*

We calculated suprathreshold (dB re threshold) wave I amplitudes at three timepoints: pre-SD, post-SD, and post-2w. As shown in Figure 2C, the baseline (pre-SD) of the ABR wave I amplitudes for all groups were all at the same level. After SD, a significant elevation in ABR wave I amplitude was observed in both of the sleep- deprived groups (1d SD and 5d SD) when compared with the control group. After 2 weeks, the ABR wave I amplitudes of the SD groups reduced to levels that were equivalent to the control group.

Then, we counted the numbers of IHC ribbons contacting auditory afferent nerve fibers by fluorescence immunohistochemistry using an antibody raised against CtBP2/RIBEYE as a metric for IHC afferent innervation at post-2w. Similar to the wave I amplitude data, we did not detect any significant differences in the ribbon count from the basal and middle turns when compared between SD groups and the control group (Figure 2D).

### Synaptopathy Caused by Over-Exposure to Noise Was Mitigated by Preceding 1d SD, but Was Aggravated by Preceding 5d SD

**FIGURE 3:**
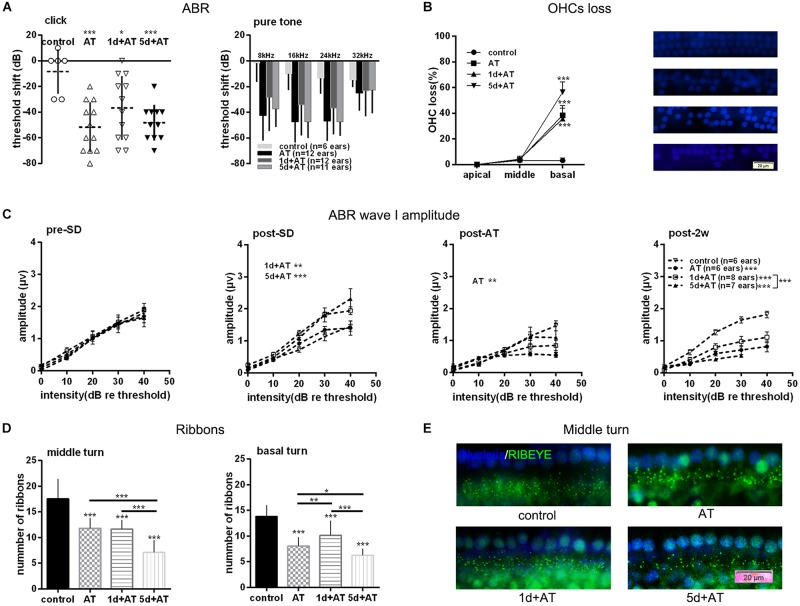
Audiometric threshold shifts, and quantification of OHCs and IHC ribbons at post-2w, and ABR wave I amplitude for noise exposed groups and the control group. **(A)** Mean (transverse line) ± S.D. and individual ear audiometric threshold shift (dB) from baseline (pre-SD) determined from click and pure tone evoked ABRs for noise exposed groups and control group. The threshold shift was statistically significantly different between noise exposed groups and control group (click: one-way ANOVA, *F* = 7.256, *P* = 0.001; pure tone: two-way ANOVA, *F* = 18.433, *P* = 0.000 for effects of AT). Among the three noise exposed groups, the threshold shift measured by tone burst was significantly different (two-way ANOVA, *F* = 3.890, *P* = 0.023 for effects of SD; AT group and 1d + AT group: *P* = 0.029; AT group and 5d + AT group: *P* = 1.000; 1d + AT group and 5d + AT group: *P* = 0.113, Table 2), but not statistically different for click stimulus (one-way ANOVA, *F* = 1.812, *P* = 0.18). **(B)** Mean ± S.D. OHCs loss percentage in the basal, middle and apical cochlear turns, and fluorescence immunohistochemistry of OHCs nucleus (blue) in the basal turn for all noise exposed groups and control group. Significant differences were observed between noise exposed groups and control group (one-way ANONA, *F* = 34.995, *P* = 0.000 for effects of AT). No significant difference was observed between AT group and 1d + AT group (*post hoc* Student’s *t*-test, *P* = 1.000) or 5d + AT group (*post hoc* Student’s *t*-test, *P* = 0.053). Significant difference was observed between the 1d + AT group and the 5d + AT group (*post hoc* Student’s *t*-test, *P* = 0.008). **(C)** Mean ± S.E.M. ABR wave I amplitude growth function for noise exposed groups and the control group. The baselines (pre-SD) of wave I amplitudes were not statistically different (two-way ANOVA, *F* = 0.089, *P* = 0.500 for effects of AT), and wave I amplitudes increased after SD (post-SD) (two-way ANOVA, *F* = 11.636, *P* = 0.000, control vs. 1d + AT group: two-way ANOVA, *P* = 0.001, control vs. 5d + AT group: two-way ANOVA, *P* = 0.000), but reduced immediately after noise exposure (two-way ANOVA, *F* = 3.388, *P* = 0.021, control vs. AT group: two-way ANOVA, *P* = 0.013). After 2 weeks, significant reduction in wave I amplitudes were observed for all AT groups (two-way ANOVA, *F* = 29.315, *P* = 0.000), and significantly differences were also observed in wave I amplitudes between 1d + AT group and 5d + AT group (two-way ANOVA, *P* = 0.000, also see Table 2). **(D)** Mean ± S.D. IHC ribbons in the basal turn and middle cochlear turn for all the AT groups at post-2w. The ribbon numbers were statistically significant different between noise exposed groups and control group for the basal turn (one-way ANONA, *F* = 69.008, *P* = 0.000) and middle turn (one-way ANONA, *F* = 76.274, *P* = 0.000). For all noise exposed groups, significant differences were observed in the basal turn between 1d + AT group and 5d + AT group (*post hoc* Student’s *t*-test, *P* = 0.000), between AT group and 1d + AT group (*post hoc* Student’s *t*-test, *P* = 0.001), or 5d + AT group (*post hoc* Student’s *t*-test, *P* = 0.032). Significant differences were also detected in the middle turn between 1d + AT group and 5d + AT group (*post hoc* Student’s *t*-test, *P* = 0.000), and between AT group and 5d + AT group (*post hoc* Student’s *t*-test, *P* = 0.000). **(E)** Fluorescence immunohistochemistry of ribbons in cochlear middle turn.

In order to explore the effects of SD on noise susceptibility, we conducted noise exposure experiments following SD. As expected, 2 h exposure to 105 dB SPL broad band noise generated a permanent threshold shift. Although there were no significant differences among the three groups exposed to noise, the mean ABR threshold shift was smaller in the 1d + AT group than in the other groups exposed to noise (Figure 3A). In order to gain a better understanding of the extent of cochlear damage, we investigated the loss of OHCs in three groups exposed to noise, and a control group (Figure 3B). All of the groups exposed to noise exhibited a significantly higher loss of OHCs in basal turns than the control group. Furthermore, the loss of OHCs in the 1d + AT group was similar to that in the AT group, although the loss of OHCs in these two groups was lower in the cochlear basal turns than that of the 5d + AT group. Not surprisingly, there were no significant differences in terms of the loss of OHCs in the middle and apical turns when compared across the three groups exposed to noise and the control group.

**TABLE 2:** *Post hoc* comparisons (Student’s *t*-tests with Bonferroni adjustment) of ABR threshold shift for pure tone stimuli, ABR wave I amplitude evoked by noise burst stimuli at post-2w.

	**8**	**16**	**24**	**32**	
**Pure tone frequency (kHz)**					
Control vs. AT	^∗∗^	^∗∗^	^∗∗^	n.s.	
Control vs. 1d + AT	n.s.	n.s.	^∗^	n.s.	
Control vs. 5d + AT	^∗∗^	^∗∗^	^∗∗^	n.s.	
AT vs. 1d + AT	n.s.	n.s.	n.s.	n.s.	
AT vs. 5d + AT	n.s.	n.s.	n.s.	n.s.	
1d + AT vs. 5d + AT	n.s.	n.s.	n.s.	n.s.	

	**0**	**10**	**20**	**30**	**40**

**Intensity (dB re threshold)**					
Control vs. AT	n.s.	^∗∗^	^∗∗^	^∗∗^	^∗∗^
Control vs. 1d + AT	n.s.	n.s.	^∗^	^∗^	^∗^
Control vs. 5d + AT	n.s.	^∗^	^∗∗∗^	^∗∗^	
AT vs. 1d + AT	n.s.	n.s.	n.s.	n.s.	n.s.
AT vs. 5d + AT	n.s.	n.s.	n.s.	n.s.	
1d + AT vs. 5d + AT	n.s.	n.s.	^∗^	n.s.	

*^∗^p < 0.05; ^∗∗^p < 0.01; ^∗∗∗^p < 0.001; n.s., not significant.*

As expected, 2 h exposure to 105 dB SPL broad band noise generated a reduction in wave I amplitude (Figure 3C). There was a significant difference between the AT group and the control group. After 2 weeks, all three groups that were exposed to noise exhibited a significant reduction in wave I amplitude, compared to the control group. Moreover, the 1d + AT group exhibited a smaller reduction in wave I amplitude than the AT and 5d + AT groups, although this difference was statistically significant.

Correspondingly, there was a significantly smaller number of IHC ribbons in the cochlear basal turn and middle turn in the AT, 1d + AT, and 5d + AT groups than in the control group (Figure 3D). In the basal turn, the reduction in the number of ribbons in all of the groups exposed to noise mirrored the reduction in wave I amplitude. The 1d + AT group had the largest number of ribbons, while the 5d + AT group had the least. In the middle turn, the 1d + AT group had a similar number of ribbons as the AT group; and was significantly higher than the 5d + AT group. Figure 3E shows fluorescence immunohistochemistry of ribbons in cochlear middle turn. Data arising relating to the ribbon number of IHC synapses, and wave I amplitude, revealed that short-term SD played a protective role. In contrast, repeated episodes of SD aggravated synaptopathy in mice exposed to noise.

### SD Treatment Exerted Temporary Influence Over Serum Corticosterone Level

**FIGURE 4:**
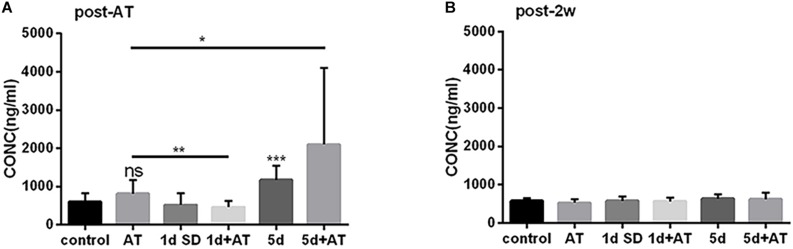
The changes in serum corticosterone concentration induced by SD. **(A)** Serum corticosterone concentration at post-AT. The corticosterone level was almost doubled in 5d SD group (*n* = 12) than that of the control group (*n* = 12). The 5d + AT group (*n* = 12) had higher corticosterone concentrations than the AT group (*n* = 12). There was no significant difference between the 1d SD group (*n* = 15) and the control group in serum corticosterone concentration. Significant reduction of serum corticosterone level was observed in the 1d + AT group (*n* = 15) as compared to the AT group (*n* = 12). **(B)** Corticosterone concentration of serum at post-2w. No significant differences of the corticosterone levels were seen among six groups at the time point of post-2w. The data are shown as the mean ± SD. Two-tailed, unpaired Student’s *t*-tests were used to determine statistical significance.

The mean concentration of corticosterone in the 5d SD group was 1173 ng/ml, significantly higher than that of the control group (600 ng/ml) (Figure 4A). However, after 1d SD, the mean concentration of corticosterone was 519 ng/ml and was not significantly different from the control group. We also determined corticosterone levels in groups exposed to noise immediately after the noise trauma. There was a dramatic elevation in the concentration of corticosterone in the 5d + AT group (2097 ng/ml). In contrast, when compared to the AT group, the 1d + AT group showed a significant reduction of corticosterone level (462 ng/ml). Notably, there were no significant differences observed between the control and AT group, between the 1d SD and 1d + AT group, or between the 5d SD and 5d + AT group. This suggested that repeated episodes of SD exerted a greater impact on serum corticosterone level than noise exposure. After 2 weeks, the levels of corticosterone in all experimental groups recovered to levels similar to the control group (Figure 4B).

### Autophagy Proteins Were Differentially Regulated by SD in Groups of Mice Exposed to Noise

**FIGURE 5:**
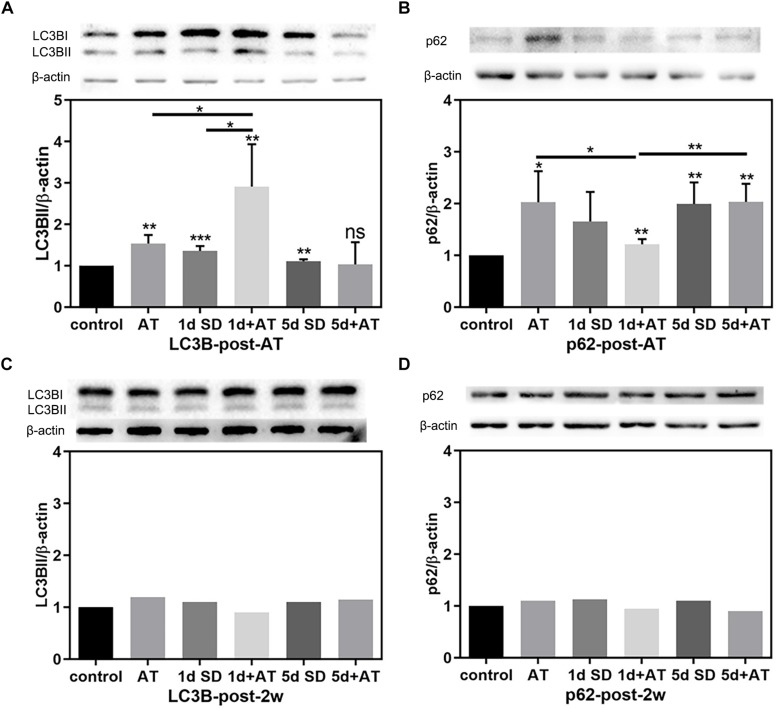
The changes in autophagy protein expression in cochleae after SD and AT procedures. **(A)** The western-blotting analysis of LC3B expression in all six groups, showing the ratios of LC3BII to β-actin after acoustic trauma (post-AT) (*n* = 4). **(B)** The western-blotting analysis of p62 expression in all six groups, showing the ratios of p62 to β-actin at the time point of post-AT (*n* = 4). **(C)** The western-blotting analysis of LC3B expression at post-2w (*n* = 1). **(D)** The western-blotting analysis of P62 expression at post-2w (*n* = 1). The data are shown as the mean ± SD. Two-tailed, unpaired Student’s *t*-tests were used to determine statistical significance.

In order to unravel the mechanisms underlying the impact of SD on noise-induced cochlear damage, we quantified the expression levels of proteins involved in cochlea autophagy by immunoblotting at two time points: post-AT and post-2w. Figures 5A,B show the expression levels of autophagy proteins in the six groups of mice at the post-AT time point. The 1d + AT group showed the most dramatic elevation in microtubule-associated protein 1 light chain 3B (LC3B); there were significant differences between the 1d + AT group and the other experimental groups (Figure 5A). In contrast, there was no apparent increase in LC3B levels after 5 days of SD preceding noise exposure. In addition, we determined that protein p62 serves as a substrate for the regulation of autophagy (Figure 5B). The expression of p62 was significantly higher in the cochleae following acoustic trauma with or without SD pretreatment, as compared with the control group. In contrast with the changes observed for LC3B, the autophagy substrate, protein p62, was expressed at the lowest levels in the 1d + AT group; this was significantly lower than the other groups of mice exposed to noise (the AT and 5d + AT groups). After 2 weeks, the expression levels of LC3B and p62 returned to levels that were similar to the control group (Figures 5C,D).

### Oxidative Stress Injury Caused an Increase of Autophagy and Apoptosis in HEI-OC-1 Cells and Cochlea HCs

**FIGURE 6:**
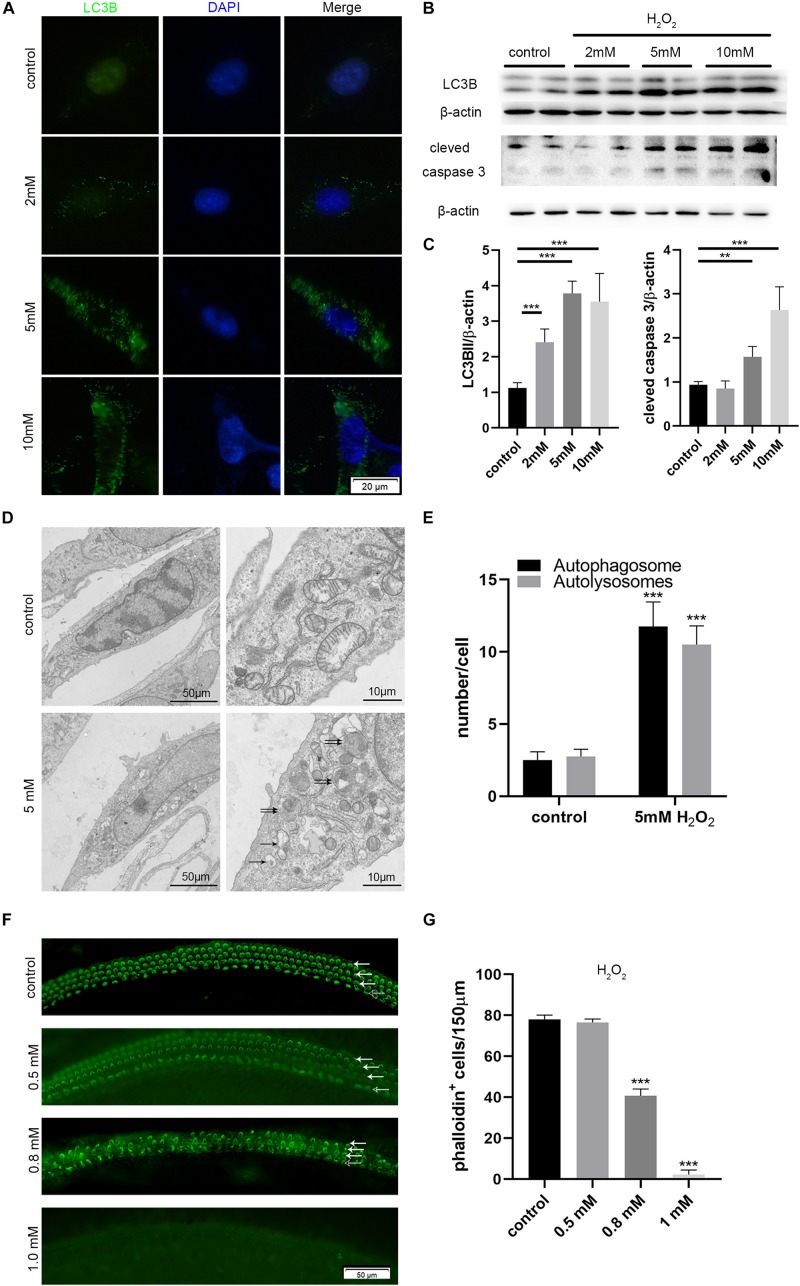
Increased autophagy and apoptosis in the HEI-OC-1 cells and cochlear hair cells (HCs) after H_2_O_2_ treatment. **(A,B)** Immunofluorescence of LC3B expression in the HEI-OC-1 cells treated with different concentrations of H_2_O_2_ (2 mM, 5 mM and 10 mM), and the western-blotting analysis of LC3B and cleaved caspase 3. **(C)** Quantification analysis of **(B)**, showing the ratios of LC3B II or cleaved caspase 3 to β-actin (*n* = 4). **(D)** Transmission electron microscope (TEM) evaluating autophagy in HEI-OC-1 cells treated with 5 mM H_2_O_2_. **(E)** Quantification of the results in **(D)**. The numbers of autophagosomes (arrow in pictures) and autolysosomes (double arrows in pictures) were significantly increased after treatment compared with the control, *n* = 3. **(F)** Phalloidin labeling showing HCs survival in the cochlea after H_2_O_2_ (0.5 mM, 0.8 mM, 1 mM) damage. OHCs, solid arrows; IHCs, blank arrows. **(G)** Quantification of the phalloidin-positive HCs in **(F)**, *n* = 3. The data are shown as the mean ± SD. Two-tailed, unpaired Student’s *t*-tests were used to determine statistical significance.

We incubated HEI-OC-1 auditory cells with different concentrations of H_2_O_2_ (2, 5, and 10 mM) for 1 h to induce oxidative stress injury. We then examined the expression of LC3B, apoptosis marker of cleaved caspase 3, and autophagy flux, after 6 h of incubation. After 24 h of incubation, we investigated cell apoptosis. Immunofluorescence and western blotting results showed that different concentrations of H_2_O_2_ caused a significant increase in the expression of LC3B and cleaved caspase 3 (Figures 6A,B). Figure 6C shows the quantification analysis of Figure 6B. TEM images further showed that the number of autophagosomes and autolysosomes was significantly higher after 5 mM H_2_O_2_ treatment, compared with the controls, thus confirming the occurrence of autophagy after oxidative stress injury (Figures 6D,E).

Then, we cultured the middle cochlea basilar membranes with different concentrations of H_2_O_2_ (0.5, 0.8, 1 mM) for 1 h and determined the HCs survival number by phalloidin-labeling after 24 h of incubation. Cell counting indicated that 0.5 mM H_2_O_2_ treatment caused 50% of HCs to die (Figures 6F,G).

### Pretreatment With Dexamethasone Increased the Levels of Apoptosis Induced by Oxidative Stress Injury in HEI-OC-1 Cells

**FIGURE 7:**
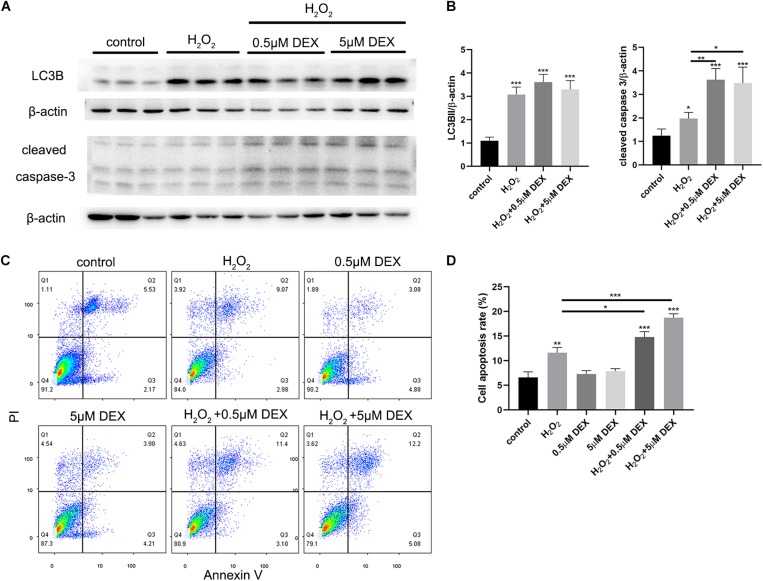
Dexamethasone (DEX) preconditioning aggravated the apoptosis induced by 5 mM H_2_O_2_ in the HEI-OC-1 cells. **(A)** Western-blotting analysis of LC3B and cleaved caspase 3 expressions. **(B)** Quantification analysis of **(A)**, showing the ratios of LC3B II or cleaved caspase 3 to β-actin, *n* = 4. **(C)** Flow cytometry for cell apoptosis analysis of different treatments. The sum of the lower right quadrants and upper right quadrants of the images represent apoptotic cells, *n* = 3. **(D)** Quantification of the flow cytometry data. The proportions of apoptotic cells after H_2_O_2_ treatment were significantly increased compared with the control group. In addition, the apoptotic proportions induced by H_2_O_2_ could be increased by dexamethasone preconditioning. The data are shown as the mean ± SD. Two-tailed, unpaired Student’s *t*-tests were used to determine statistical significance.

HEI-OC-1 cells were treated with different concentrations of dexamethasone (0.5, 5 μM) for 24 h, and then incubated with 5 mM H_2_O_2_ for 1 h. The expression levels of LC3B, and cleaved caspase 3, were then quantified after 6 h of incubation. We also investigated the extent of cellular apoptosis after 24 h. Western blots showed that dexamethasone pretreatment significantly increased the expression of cleaved caspase 3 induced by H_2_O_2_ (Figure 7A), although there was no significant difference in terms of LC3B expression levels. Figure 7B shows the quantification analysis of Figure 7A. We also used propidium iodide and ANXA5/Annexin V labeling to investigate apoptosis at 24 h after H_2_O_2_ and dexamethasone treatment. Following H_2_O_2_ treatment, the proportion of cells that was undergoing apoptosis was 11.6% (Figures 7C,D). Combining the treatments with different concentrations of dexamethasone (0.5 and 5 μM) and H_2_O_2_ treatment, we found that the proportion of apoptotic cells had increased significantly to 14.8 and 18.7%, respectively. The proportion of cells in the dexamethasone treatment groups that was undergoing apoptosis was not significantly different than the control group.

### Rapamycin Treatment Partly Rescued Apoptosis in HEI-OC-1 Cells and the Loss of HCs Induced by H_2_O_2_

**FIGURE 8:**
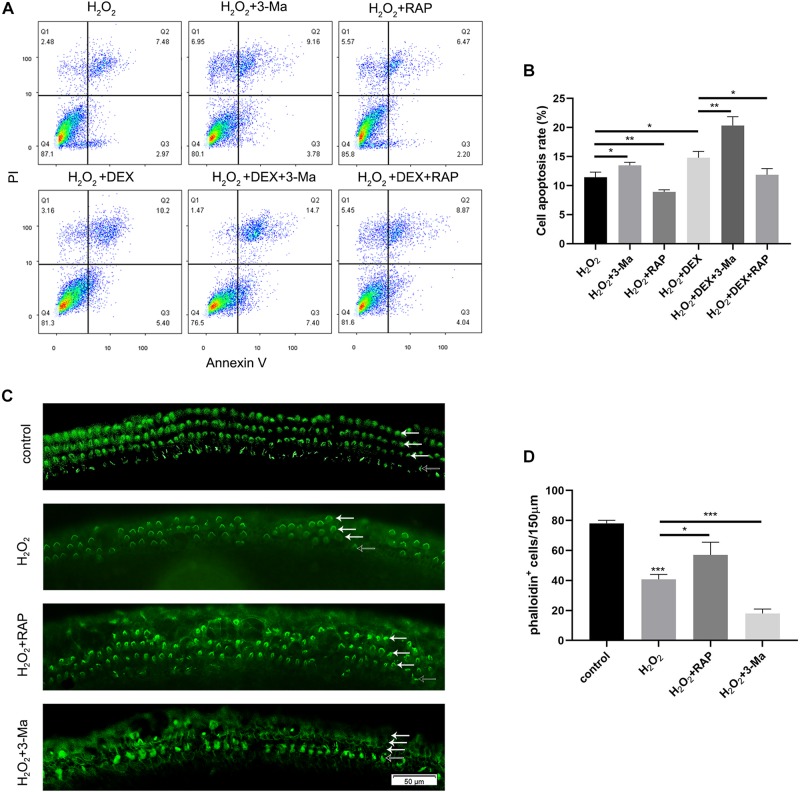
Autophagy affected the apoptosis in HEI-OC-1 cells and the loss of hair cells (HCs) induced by H_2_O_2_ (5 mM, 0.8 mM, respectively) exposure. **(A)** Flow cytometry for cell apoptosis analysis of different treatments, *n* = 3. **(B)** Quantification of the flow cytometry data of **(A)**. The proportions of apoptotic cells could be increased by 3-MA and decreased by rapamycin. Rapamycin treatment partly prevented the increased HEI-OC-1 cells apoptosis induced by dexamethasone and H_2_O_2_. **(C)** Phalloidin labeling showed HCs survival in the cochleae after H_2_O_2_ damage with 3-MA or rapamycin preconditioning. **(D)** Quantification of the phalloidin-positive HCs in **(C)**, *n* = 3. The HCs loss induced by H_2_O_2_ were partly prevented by rapamycin treatment but aggravated by 3-MA treatment. The data are shown as the mean ± SD. Two-tailed, unpaired Student’s *t*-tests were used to determine statistical significance.

To determine whether autophagy plays a role in oxidative stress injury, we pretreated HEI-OC-1 cells with 0.5 μM dexamethasone for 24 h, 0.5 nM rapamycin (RAP), or 5 mM 3-Ma, for 6 h before exposure to H_2_O_2_. The cells were then treated with 5 mM H_2_O_2_ for 1 h and allowed to recover in culture medium for another 24 h, together with dexamethasone, rapamycin, or 3-MA. Cellular apoptosis was then detected by propidium iodide and ANXA5/Annexin V labeling. We found that the proportion of apoptotic cells in the H_2_O_2_ + RAP group was significantly reduced when compared with the H_2_O_2_ group (Figures 8A,B). Correspondingly, 3-Ma treatment increased the level of apoptosis induced by H_2_O_2_ injury. Pretreatment with dexamethasone and 3-Ma had cumulative effects on apoptosis. We found that the apoptotic ratio of the H_2_O_2_ + DEX + 3-Ma group was significantly higher than that of the H_2_O_2_ + DEX and H_2_O_2_ + 3-Ma, groups. In summary, we found that H_2_O_2_-induced apoptosis in HEI-OC-1 cells and that this was further enhanced by dexamethasone. However, rapamycin treatment rescued this effect on apoptosis and reduced the apoptotic ratio from 14.8 to 11.8%.

Finally, we investigated the survival of HCs after pretreatment with 0.5nM rapamycin, and subsequent treatment with 0.8 mM H_2_O_2_, by counting the number of phalloidin-positive HCs. Compared to the H_2_O_2_-only group, HCs had a higher survival rate in the H_2_O_2_ + RAP group, and showed a higher mortality rate in the H_2_O_2_ + 3-Ma group (Figures 8C,D).

## Discussion

In this study, we used a mouse model to demonstrate that noise-induced synaptopathy varied with the duration of sleep deprivation (SD), but also that the noise-induced hearing threshold shift did not change with the duration of SD. Synaptopathy was indicated by the reduced numbers of synaptic ribbons and the reduced wave I amplitude. In addition, the extent of autophagy (based on the levels of LC3B and p62) was mildly increased following acoustic trauma (AT) alone, but significantly increased after short-term SD (1d SD) followed by AT, and remained at control levels after repeated episodes of SD (5d SD) followed by AT. Similarly, the levels of corticosterone varied with the duration of SD. Compared to AT alone, the levels of corticosterone were mildly reduced after 1d SD + AT, but significantly increased after 5d SD + AT. *In vitro* experiments further indicated that a high concentration of dexamethasone, and the inhibition of autophagy, increased the level of apoptosis induced by H_2_O_2_, and that the up-regulation of autophagy-enhanced hair cell protection. It is also important to note that repeated episodes of SD aggravated noise-induced synaptopathy. We consider that these negative effects not only occurred in SD mice exposed to high-intensity laboratory noise, but also in people with sleep disorders who experience noise exposure during their life. However, these negative effects are likely to be ignored in traditional audiometric methods. Meanwhile, the accumulation of these negative effects may contribute to inner ear disease, including tinnitus and sudden deafness. Admittedly, our study only featured female mice; it is therefore difficult to extrapolate whether our results may have differed if we had used male mice. Previous studies have shown that females are more vulnerable to stress-related neuropsychiatric diseases (Palanza, 2001). This represents a potential limitation for our study.

### SD-Alone Enhanced Wave I Amplitude in a Manner That Was Independent From Corticosterone Level

We chose to investigate SD stress because many patients with auditory disorders are known to suffer from a lack of sleep. We chose corticosterone because this represents the most effective and commonly used indicator of this general stress response (Wang and Liberman, 2002; Irwin, 2015).

The levels of corticosterone in short-term sleep deprived mice exhibited a mild reduction; these results were consistent with those described in previous studies (Brianza-Padilla et al., 2018). The corticosterone levels of successively sleep-deprived mice has also been reported to increase significantly (Gonzalez-Castañeda et al., 2016; Brianza-Padilla et al., 2018). We also found that the ABR wave I amplitude was temporarily elevated following SD. Following a period of stress, high levels of corticosteroids quickly and reversibly enhance hippocampal and basolateral amygdala glutamatergic transmission via non-genomic or genomic actions (Boudaba and Tasker, 2006; Karst et al., 2010; Groeneweg et al., 2011). This may be similar to the way in which corticosterone enhanced glutamatergic release from IHCs ribbons. Thus, it was not difficult to explain the co-existence of wave I amplitude elevation, and the increase in corticosterone level, for mice experiencing repeated episodes of SD. However, for short-term SD mice, the wave I amplitude increased before the corticosterone elevation induced by SD. A recent study reported a similar finding in rats; in the control group, rats with low levels of corticosterone exhibited a high wave I amplitude, and that rats with high levels of corticosterone presented with a low wave I amplitude (Singer et al., 2018). Although there have been many related studies, it is difficult to determine the precise relationship between the level of corticosterone, or ABR wave I amplitude, and sleep deprivation. Future research should investigate the relationship between wave I amplitude and corticosterone level after SD treatment by applying glucocorticoid receptor inhibitors and agonists.

### SD Modified Noise-Induced Cochlear Synaptopathy in Relation to Variation in Stress Hormones

We observed that SD altered the levels of circulating corticosterone in mature female BALB/C mice, as well as permanent noise-induced cochlear synaptopathy. In comparison to mice exposed to AT alone, short-term SD mice exhibited an alleviation of noise-induced cochlear synaptopathy after 2 weeks. This protective effect was accompanied by low levels of corticosterone. However, this level of protection disappeared if AT occurred after repeated episodes of SD or when corticosterone levels were elevated. Based on these results, we speculated that there may be a correlation between corticosterone level and noise-induced synaptopathy. Previous studies reported that a high level of corticosterone induced by moderate levels of long-term sound (Yoshida and Liberman, 2000) or restraint stress triggered a protective effect on the cochlea (Wang and Liberman, 2002; Tahera et al., 2006; Meltser et al., 2009). These previous studies focused on the shift in hearing threshold, and the protective mechanism of OHC, in a short-term process, lasting from several hours to 1 week. A recent study switched the research focus to wave I amplitude and the loss of synaptic ribbons; the authors involved reported that high levels of corticosterone had a negative effect on noise-induced synaptopathy, and that a glucocorticoid antagonist could eliminate this effect (Singer et al., 2018). Thus, the destructive effect of stress hormones on IHC ribbons might coexist with their protective functions, that seek to reduce the vulnerability of OHCs. The results of our *in vitro* experiments indicated that high concentrations of dexamethasone alone had no effect on apoptosis, but aggravated the damage caused by H_2_O_2_. This indicated that the protection of hair cells against oxidative stress was weakened after long-term and high-concentration dexamethasone pretreatment. This suggested that corticosterone level was associated with the severe loss of OHCs induced by AT after repeated episodes of SD.

However, this seems to contradict the clinical application of synthetic glucocorticoids in the treatment of reversible hearing loss and the maintenance of hearing. In fact, a previous study showed that after the withdrawal of glucocorticoid, hearing improvement was not maintained (Meltser and Canlon, 2011). Similarly, we found that the temporarily increased levels of corticosterone were associated with temporarily enhanced auditory responses in mice treated with repeated episodes of SD. However, when corticosterone levels returned to normal, the auditory response worsened after 2 weeks. Since the model of SD was complex, we cannot simply use a glucocorticoid agonist, or antagonist, to explore the relationship between corticosterone level and auditory response. This represents another limitation of our study. Our future studies will investigate the long-term effects of glucocorticoids alone on noise-induced auditory injury.

### The Association Between SD Modified Noise-Induced Cochlear Synaptopathy and Variations in Autophagy

The activation of autophagy has been identified as a protective mechanism to prevent noise-induced injury to the OHCs (Yuan et al., 2015; Ye et al., 2019). In addition, SD is known to interfere with the autophagy-lysosomal system. Therefore, we studied the variation in autophagy in cochlea basilar membranes.

We found that SD elevated the expression of the autophagy proteins, LC3B, and p62, regardless of the duration of SD. The short-term SD followed by AT group showed higher levels of LC3B, and lower levels of p62, than the short-term SD alone group, while the repeated SD followed by AT group, showed no significant differences when compared with the repeated SD alone group. Meanwhile, at the cochlea basal turn, repeated SD + AT mice showed more synaptic ribbons and loss of OHCs than other AT mice. In addition, repeated SD + AT mice showed the co-existence of the high levels of corticosterone and the significant loss of OHCs. One potential reason for this might be that repeated SD disrupted homeostasis, accompanied by disorders in a variety of defense mechanisms, including immunological malfunction (Chao et al., 2011; Irwin, 2015) and autophagy system disorder. Short-term SD + AT was shown to activate autophagy, but not repeated SD + AT. This suggested that repeated episodes of SD disturbed the normal regulation of autophagy (Mônico-Neto et al., 2015; Cao et al., 2019). Previous neuronal studies indicated that the impairment of autophagy function led to the accumulation of misfolded and damaged proteins, thus reducing synaptic transmission efficiency and the acceleration of neurodegeneration (Vijayan and Verstreken, 2017; Liang and Sigrist, 2018; Hoffmann et al., 2019). Since the function of IHC synaptic ribbons is similar to that of the neuron presynaptic boutons, it could be inferred that a highly regulated clearance system is vital for maintaining the integrity and function of the IHC synaptic ribbon. *In vitro* experimental results further showed that oxidative stress activated autophagy in a very effective manner. Moreover, we found that variation in autophagy level caused significant effect on apoptotic processes induced by oxidative stress. The activation of autophagy caused a reduction in apoptosis, whereas the inhibition of autophagy enhanced apoptosis. This study indicated that autophagy played an important role in oxidative stress-induced injury. However, the causal relationship between autophagy and noise-induced cochlear synaptopathy has still to be investigated. Such research should uncover the effect of autophagy on synapse integrity or synaptic function in IHCs.

## Conclusion

In summary, this study demonstrated that short-term sleep deprivation exerts positive effects on noise-induced cochlea injury, and that repeated episodes of sleep deprivation aggravates this form of injury. Our *in vitro* studies also revealed that the mechanism underlying this observation involved stress hormones and autophagy. This study provides a theoretical basis for further studying the relationship between sleep deprivation and auditory trauma, and also provides a better explanation for patients who experience both sleep disorders and hearing disease.

## Data Availability Statement

The datasets generated for this study are available on request to the corresponding author.

## Ethics Statement

The animal study was reviewed and approved by the Animal Research and Ethics Committee of Tongji Medical College, Huazhong University of Science and Technology.

## Author Contributions

DB and HC conceived and designed the experiments. PL, JC, ZD, YS, and FQ performed the experiments. PL was responsible for animal models and wrote the manuscript. JC performed tissue preparation and immunohistochemistry. ZD and YZ were mainly responsible for cell culture. SW performed the TEM experiment. DB and HC interpreted the data and revised the manuscript.

## Conflict of Interest

The authors declare that the research was conducted in the absence of any commercial or financial relationships that could be construed as a potential conflict of interest.

## Abbreviations

ABR: auditory brainstem response
AT: acoustic trauma
DEX: dexamethasone
HCs: hair cells
IHCs: inner hair cells
OHCs: out hair cells
RAP: rapamycin
SD: sleep deprivation.
